# Comparative Study of Protein Expression Levels of Five Plaque Biomarkers and Relation with Carotid Plaque Type Classification in Patients after Carotid Endarterectomy

**DOI:** 10.1155/2018/4305781

**Published:** 2018-11-19

**Authors:** Konstantinos Kyriakidis, Pavlos Antoniadis, Sohail Choksy, Rigini M. Papi

**Affiliations:** ^1^Department of Vascular Surgery, Konstantopoulio General Hospital, “Agia Olga”, Athens, Greece; ^2^Colchester and Ipswich Hospital University Foundation Trust, Colchester, Essex, UK; ^3^Laboratory of Biochemistry, Department of Chemistry, Aristotle University of Thessaloniki, 54124 Thessaloniki, Greece

## Abstract

Atherosclerosis is an inflammatory process resulting in local plaque deposition in the vessel wall of arteries with symptoms to various areas of vascular tree. Identification of patients with progressive advanced atherosclerotic disease is mainly based on the known characteristics of the vulnerable or recently ruptured plaque. Molecular and cellular features associated with the vulnerable plaque are considered potential diagnostic markers for plaque rupture and thrombosis. Here, protein expression levels of the metalloproteases MMP-1, MMP-9, osteopontin (OPN), and cytokines TNF*α* and IL-6 in tissue extracts of carotid plaques in patients after endarterectomy were estimated by Western immunoblotting, after SDS-PAGE analysis and evaluated based on the ultrasonographic plaque morphology. The gender and age effect was also examined. MMP-1, MMP-9, and IL-6 were expressed in higher levels compared to OPN and TNFa as well as in symptomatic (with type II and III carotid plaque classification) than asymptomatic (type IV) patients with differences considered statistically significant (P values <0.05). A significant positive correlation between MMP-1 and IL-6 (with Pearson correlation coefficient 0.748) is also notable. The data give further insight into the possible role of specific biomarker and enhance the need for further studies in order to clarify the proper one(s) for detection of the vulnerable plaque and help identify patients at risk for cardiovascular events.

## 1. Introduction

Atherosclerosis is a chronic, inflammatory disease of the medium and large arteries, such as the aorta and carotid arteries. It is a major contributor to the development of cardiovascular diseases and the leading cause of death worldwide. Although atherosclerotic plaque development is a local process in the vessel wall that can give rise to symptoms in one specific area, it is also a systemic disease with simultaneous plaque formation in different areas of the vasculature [1–3]. Moreover, studies have shown that atherosclerotic carotid arteries pose a substantial risk of ipsilateral cerebrovascular events, with reported annual ischemic stroke rates ranging from 0.35% to 1.3% in asymptomatic patients with moderate stenosis [4, 5] and from 0.5% up to 5% for severe asymptomatic carotid artery stenosis [5, 6].

Atherosclerotic plaques are characterized by intimal thickening from the progressive accumulation of lipids [1, 2] together with other cellular and molecular components such as smooth muscle cells, monocytes, T cells, B lymphocytes, erythrocytes, and platelets. These cells are able to produce and secrete mediator molecules such as cytokines, chemokines, growth-factors, enzymes, and disintegrins, which activate endothelial cells, proliferation of smooth muscle cells, and lesion progression, and contribute to the weakening of a vulnerable plaque by matrix degradation of its fibrous cap. Many of these involved molecules can be measured systemically, and it has been shown that elevated concentrations in the circulation are associated with future cardiovascular events.

In other reports, for example, serum markers such as high-sensitive C-reactive protein, interleukin-6 [7–9], and IL-18 [10] are correlated with the development, progression, and rupture of atherosclerotic plaque [2, 11]. It is also suggested that inflammatory markers are transcribed locally in atherosclerotic plaques [12–15].

Other markers that have been previously associated with cardiovascular events and are now associated with carotid atherosclerotic plaques are matrix metalloproteinases (MMPs) [16], tissue inhibitor of metalloproteases (TIMP) [17], soluble intercellular adhesion molecule 1 [18], and osteopontin [14, 19]. MMPs are a family of zinc-containing enzymes and are secreted as inactive precursors; they are prevalent in the arterial wall throughout the arterial system and play a central role in degradation of the vascular extracellular matrix resulting in destabilization of the atherosclerotic plaque [16]. The destabilization event contributes to plaque rupture and consequently in acute ischaemic events. Increased carotid MMP-9 plaque levels are associated with an unstable plaque phenotype and are higher in lipid-rich inflammatory plaques. Osteopontin, an acidic phosphoprotein, has recently been demonstrated to inhibit mineral deposition as well as osteoclastogenesis and is constitutively expressed by a wide range of cell types in the vasculature. Carotid plaque contains valuable information for follow-up after vascular surgery. It has been shown that local plaque characteristics are associated with restenosis at the site of carotid endarterectomy after 1 year and endarterectomy of lipid-rich, inflammatory plaques, associated with reduced risk of restenosis compared to stable, fibrous plaques, independent from clinical characteristics [20].

It has been also reported that carotid plaque composition contains predictive information for future cardiovascular events elsewhere in the vascular system, independent from established risk factors and medication. Moreover, patients undergoing carotid endarterectomy, with a local plaque containing intra plaque hemorrhage or marked intra plaque micro vessel formation, demonstrated an increased risk of secondary cardiovascular events with high hazard ratios [21, 22]. Thus, determination of these molecules may give important prognostic information and may in turn be useful in improving risk stratification. However, for most of these biomarkers the clinical utility and their specificity have not yet been established.

This study looks at evaluating carotid plaques in symptomatic and asymptomatic patients after endarterectomy in order to potentially detect vulnerable plaques, based on the differences in protein expression levels of five biomarkers.

## 2. Material and Methods

### 2.1. Materials

Rabbit polyclonal antibodies that recognise MMP-1, MMP-9, IL-6, and TNF*α* were purchased from Acris Antibodies (GmbH, Germany), while anti-osteopontin was purchased from Millipore. The rabbit polyclonal antibody raised against actin and the secondary anti-rabbit antibody conjugated with alkaline phosphatase were purchased from Sigma-Aldrich Chem Co (St. Louis, MO, USA). All other chemicals used in the study were also purchased from Sigma-Aldrich.

### 2.2. Subjects

#### 2.2.1. Carotid Atherosclerotic Plaque Sample Preparation

24 patients (14 males, 10 females) age (55-85) were enrolled from the Konstantopoulio General Hospital, Department of Vascular Surgery, Athens, Greece, who had undergone standard carotid ultrasound examination and subsequently carotid endarterectomy. All patients were entered into a prospective database and were on best medical treatment. Colour Doppler ultrasonography and Power Doppler were used in addition to serial ultrasonographic measurements, in order to monitor and evaluate the carotid plaque type and vulnerability preoperatively. Following the carotid endarterectomy, the carotid plaque specimen from each patient was immediately washed and stored at -80°C under liquid nitrogen, until tissue homogenization and subsequent analysis.

The criteria to perform a carotid endarterectomy are based on the recommendations by the ACAS study [23] (Toole JF, 1996) for asymptomatic patients and the NASCET study [24] for symptomatic patients. Sixteen samples (Table 1) were obtained from carotids of symptomatic patients with carotid plaques II and III, and eight samples** (**Table 1) from asymptomatic patients, all of which had carotid plaque type IV (Table 1). At baseline, clinical parameters, including cardiovascular risk factors and medication used, were recorded. Patients were assigned a unique number in chronological order based on the time of surgical operation of each patient, which is used to identify the samples.

#### 2.2.2. Carotid Ultrasonography

All ultrasound examinations were performed with a Phillips SONOS 5500 equipped with a 7.5MHz linear-array transducer. Initially, the common and internal carotid arteries were scanned in cross-section and longitudinally by B-mode and color Doppler methods, through which the largest plaque was identified for evaluation of plaque echogenicity. Plaque thickness was measured on the longitudinal B-mode or color Doppler images perpendicular to the vascular wall.

#### 2.2.3. Protein Extraction

Frozen carotid plaque samples were homogenized in 50 mM Hepes pH 7.5, 150 mM NaCl, 15 mM *β*-mercaptoethanol, 0.5 mM phenylmethylsulfonyl fluoride, and 0.1% (v/v) Nonidet P-40 according to Sigala et al. [25]. The homogenates were centrifuged at 2000g for 10 min to obtain total protein extracts that were subsequently assessed by western immunoblotting. Protein concentration in tissue extracts was determined by the Bradford method [26], using bovine serum albumin as the reference standard.

#### 2.2.4. Electrophoresis and Immunoblotting Analysis

Proteins were separated by SDS polyacrylamide gel electrophoresis (SDS-PAGE) using 10% (w/v) polyacrylamide gels containing 0.1% (w/v) SDS as described by Laemmli et al. [27]. Proteins were either stained with Coomassie Brilliant Blue R250 or electrotransferred to nitrocellulose membranes as described by Towbin et al. [28]. Prestained protein markers were used for identification. The immunoblotting analysis was performed using commercially available antibodies raised against MMP-1, MMP-9, IL-6, TNF*α* (Acris), and OPN** (**Millipore**)**. The anti-actin antibody (Sigma-Aldrich) was used for assessing equal loading of total protein per sample. Gel-Pro Analyzer was used to evaluate and record the intensity of each band.

#### 2.2.5. Statistical Analysis

Statistical analysis was performed by SPSS (version 23) software and Microsoft Excel 2011 for Mac and the data were expressed as mean values ± standard deviation (SD). Independent samples T-test was used to determine differences in the variables. P values less than 0.05 were considered significant at confidence level 95%. Pearson correlation analysis was used to assess correlation between the biomarkers.

## 3. Results and Discussion

**Figure 1:**
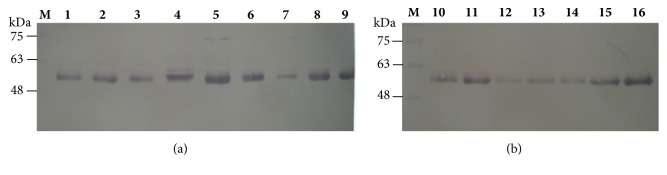
Western blot analysis of proteins using MMP-1 antibody. Proteins were analysed on 10% w/v SDS-polyacrylamide gels. M: prestained protein markers, lanes 1 -9 (a), 10-16 (b): 50 *μ*g total proteins.

**Figure 2:**
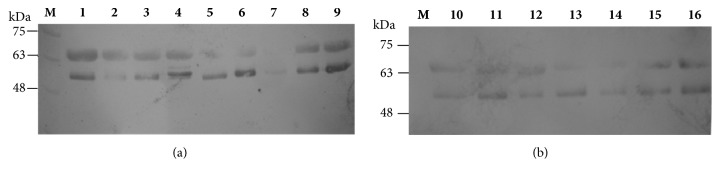
Western blot analysis of proteins using MMP-9 antibody. Proteins were analysed on 10% w/v SDS-polyacrylamide gels. M: prestained protein markers, lanes 1-9 (a) and 10-16 (b): 50 *μ*g total proteins.

**Figure 3:**
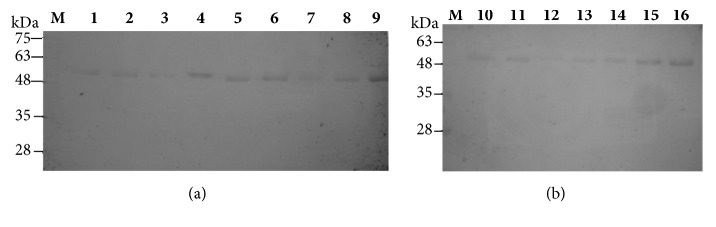
Western blot analysis of protein expression using IL-6 antibody. Proteins were analysed on 10% w/v SDS-polyacrylamide gels. M: prestained protein markers, lanes 1-9 (a) and 10 -16 (b): 50 *μ*g total proteins.

Carotid duplex ultrasound provides quantitative measurements of carotid artery stenosis and intima-media thickness. The diagnosis of carotid artery stenosis is important in clinical practice because it determines the need for surgical intervention while carotid intima-media thickness measurements provide a quantitative measure of disease [29]. This study included 24 patients identified as symptomatic (n=16) or asymptomatic (n=8) following a Doppler scan who then went on to have a carotid endarterectomy. Plaque type II was recorded in 9 out of 16 symptomatic patients with prevalence of men, 7 out of 9. Table 1 lists the patients' demographic and clinical characteristics. The carotid plaques were assessed for the expression levels of the metalloproteases; MMP-1, MMP-9, and OPN and for cytokines TNFa and IL-6. Total proteins of the carotid plaques samples were separated by SDS-PAGE analysis as reported above, followed by Western blotting analysis using MMP-1, MMP-9, IL-6, TNFa, and OPN antibodies. Proteins were analysed on 10% (w/v) SDS-polyacrylamide gels. Prestained protein markers were used for identification. Indicatively, lanes 1-9 (a) and 10-16 (b) of samples 1 to 16 for MMP-1, MMP-9, and IL-6 are shown in Figures 1, 2, and 3, respectively. MMP-9 expression in the tissue extracts gave two bands of lower molecular weight in all samples, symptomatic and asymptomatic, instead of the expected one band at 83 kDa for MMP-9 or at 92 kDa for proMMP-9 showing degradation of MMP-9 in the carotid extracts. On the other hand, IL-6 in the tissue extracts of study samples was detected as a dimer (Figure 3).

**Figure 4:**
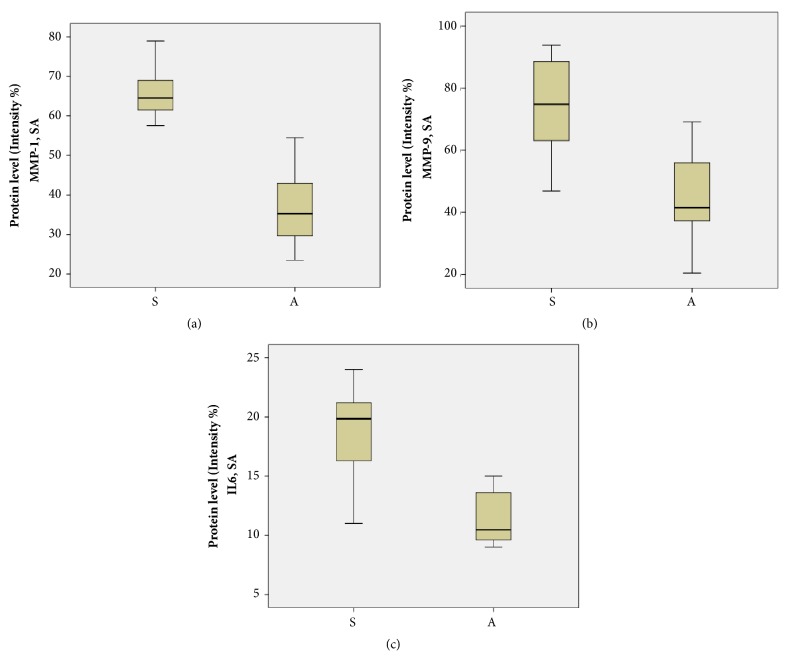
Boxplot depiction of differences in MMP-1 (a), (MMP-9) (b) and IL-6 (c) values between symptomatic and asymptomatic patients (P=0).

Statistical significant differences in MMP-1 levels between symptomatic and asymptomatic patients, P<0.05 (P=0), were recorded (Figure 4(a)).

Higher protein levels of MMP-9 were also detected in the samples of symptomatic than in asymptomatic patients (Figure 4(b)), with the differences being statistically significant P<0.05. Our data are in agreement with previous studies showing that MMPs, including MMP-1, MMP-3, and MMP-9, were overexpressed in human atherosclerotic plaques and in animal models and this is particularly associated with macrophages [30]. Elevated levels of IL-6 were observed more prominently in symptomatic (with plaque type II/III) versus asymptomatic patients (plaque type IV) as can be seen in Figure 4(c); the observed differences are statistically significant (P=0). Furthermore, studies of functional genetic polymorphisms in genes encoding five MMPs (MMP-1, MMP-3, MMP-7, MMP-9, and MMP-12) in relation to subclinical atherosclerosis phenotypes revealed a strong association of MMP-9 279Q allele and the presence of plaques in men and specifically carotid plaques [31]. A gender-specific effect of another MMP-9 functional polymorphism and carotid arterial stiffness was also reported by Lin et al. [32]. Here, no significant gender effect is found, in symptomatic and asymptomatic samples. However, a larger population with sample variety in addition to simultaneously studying the impact of genetic variation and protein expression level of MMP-9 is a study we plan to conduct.

The expression level of OPN was low and the variation within samples from symptomatic and asymptomatic patients was statistically insignificant at a confidence level 95%, with P=0.2. Previous reports show that carotid plaque osteopontin level is a predictor of cardiovascular events after carotid endarterectomy [33] and high levels of it recorded in serum [34]. On the other hand, lower OPN levels result in mineral deposition and plaque calcification and, therefore, enhance plaque stability and decrease the likelihood of clinical events [35].

Cytokines are key regulatory glycoproteins that modulate all aspects of vascular inflammation. Many cytokines have been implicated in atheroma formation and complication. In this study, the cytokines IL-6 and TNFa were measured. IL-6 is a phosphoglycoprotein with MW ranging from 21 to 29 kDa, depending on the degree of glycosylation and phosphorylation and IL-6 dimer forms a complex with the dimer of IL-6 receptor and the dimer of gp-130 to initiate signal transduction [36, 37]. IL-6 enhances cell adhesion molecule expression as well as the production of acute phase reactants such as CRP and TNFa by the hepatocytes. Higher IL-6 levels are associated with lower echogenicity of carotid plaques suggesting a link between inflammation and potential risk of plaques [38]. Epidemiological studies have found increased vascular risk in association with increased basal levels of cytokines IL-6 and TNFa, cell adhesion molecules such as soluble ICAM-1, P selectin, E selectin, and downstream acute-phase reactants such as CRP, fibrinogen, and serum amyloid A [39]. The proinflammatory protein levels, TNFa, are also related to carotid atherosclerosis [40]. Here, TNFa was detected as a dimer, as in the case of IL-6, though expressed at low yields in all samples and no statistically significant differences in the mean values between symptomatic and asymptomatic samples were observed, with P=0.09 at a confidence level 95%** (**data not shown).

**Figure 5:**
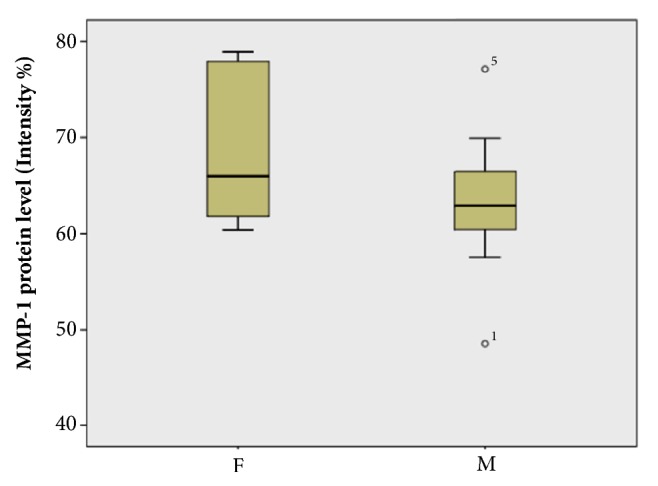
Boxplot depiction of MMP-1 values of male and female symptomatic patients (P>.05).

**Figure 6:**
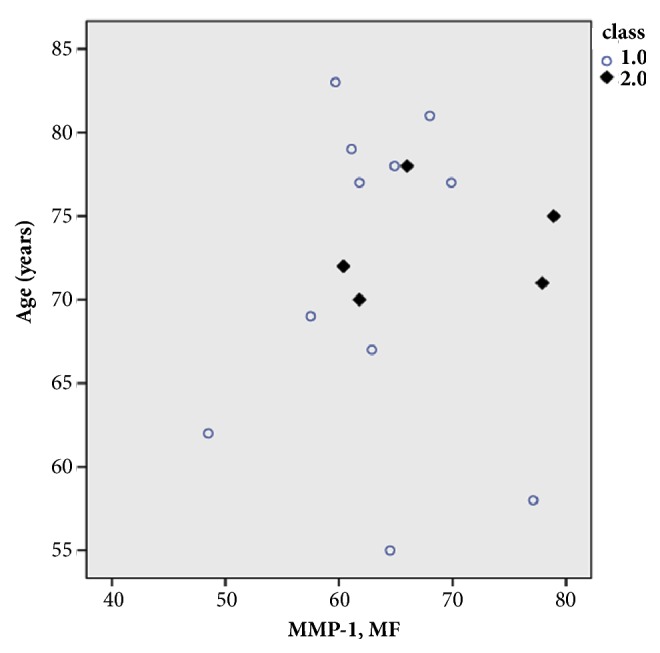
Scatterplot analysis of MMP-1 levels in male (○) and female (♦) versus age in symptomatic patients.

Expression levels of these five biomarkers, both in symptomatic and asymptomatic patients, were also evaluated regarding the gender of patients. There is no statistical significant difference between males (n=11) and females (n=5) in the values of protein expression levels in all cases of symptomatic patients. The boxplot of the mean MMP-1 values in male and female symptomatic patients, indicatively, is shown in Figure 5. Gender differences in asymptomatic patients with atherosclerotic carotid plaques are reported by Ota et al. [41]. Furthermore, no significant differences were recorded in the expression levels of the biomarkers versus the age of patients both symptomatic and asymptomatic ones. The effect of age (in the range of 55-85 years) on MMP-1 values, indicatively, of male (MMP-1M) and female (MMP-1F) symptomatic patients is shown in the scatterplot of Figure 6. Similar studies were also conducted with the other four biomarkers (data not shown). It can be said that in patients over 70 years of age, the values of the expression levels of all biomarkers and specifically of MMP-1, MMP-9, and IL-6 are elevated and the differences between male and female values tend to coincide. This is in agreement with previous report indicating that there is no age effect [42].

**Figure 7:**
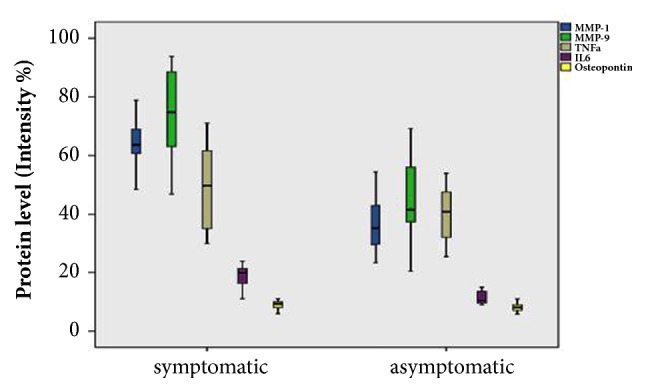
Boxplot of the mean values of the protein levels of all the biomarkers for symptomatic and asymptomatic patients.

Pearson correlation values were used to examine linear correlation between the biomarkers. Pearson correlation coefficient value of 0.748 for MMP-1 and IL-6 and 0.606 for MMP-9 and IL-6 (Table 2) indicates high levels of correlation between them, while that of 0.594 for MMP-1 and MMP-9 indicates a moderate level. Values are significant in the 0.01 level (two-tailed). The differences in mean values of protein expression levels of the five biomarkers between symptomatic and asymptomatic patients are shown in the box plot in Figure 7. Further attempt to correlate the levels of the biomarkers in symptomatic patients, with the specific plaque types II and III, did not show any clear association, if there is such (data not shown), due to the moderate number of samples included in each case.

## 4. Conclusion

The results demonstrate that expression levels of MMP-1, MMP-9, and IL-6 were significantly more pronounced than OPN and TNFa, respectively, and statistical significant differences in the values of the first three between symptomatic and asymptomatic patients were also observed. The findings support that elevated levels of the three biomarkers identify symptomatic patients and may be used for vulnerable plaque classification and the prediction of patients' risk for cardiovascular events.

It is worth mentioning that despite the prominent differences in the protein expression level of the biomarkers studied, no control samples from healthy individuals could be used in order to have a threshold value to compare with, indicating that a standard control when evaluating a biomarker is still of major concern. This is due to obvious ethical issues and carotid plaque analysis is therefore limited to patients who undergo vascular surgery.

Thus, these data analyses prompt further studies, evaluating larger groups of patients and compare the protein expression levels of the biomarkers in carotid plaques versus the circulating ones which may give rise to tissue specific biomarkers. Therefore, it may provide evidence towards patient selection and the treatment planning in order to decrease the perioperative risk.

## Figures and Tables

**Table 1:** Patient's demographic, clinical characteristics and plaque morphology.

**Subject No**	**Gender**	**Age**	**Symptomatic**	**Asymptomatic**	**Carotid plaque type**
1	M	62	+		II

2	M	69	+		II

3	M	67		+	IV

4	M	78	+		II

5	M	77	+		II

6	F	75	+		III

7	F	74		+	IV

8	F	71	+		III

9	M	58	+		II

10	F	71		+	IV

11	M	79	+		III

12	F	70		+	IV

13	M	61		+	IV

14	F	69		+	IV

15	F	72	+		II

16	M	81	+		III

17	M	83	+		III

18	F	70	+		III

19	F	66		+	IV

20	M	73		+	IV

21	M	77	+		III

22	M	67	+		II

23	M	55	+		II

24	F	78	+		II

**Table 2:** Pearson correlation coefficient values between the biomarkers.

	MM-1	MMP-9	TNFa	IL-6	Osteopontin
MMP-1	1				

MMP-9	0.594^a^	1			

TNFa	0.164	0.399	1		

IL-6	0.748^a^	0.606^a^	0.172	1	

Osteopontin	0.365	0.476^b^	0.469^b^	0.116	1

^a^Correlation is significant at the 0.01 level (2-tailed)

^b^Correlation is significant at the 0.05 level (2-tailed)

## Data Availability

The data used to support the findings of this study are available from the corresponding author upon request.

## References

[1] Ross R. (1999). Atherosclerosis—an inflammatory disease. *The New England Journal of Medicine*.

[2] Libby P., Ridker P. M., Maseri A. (2002). Inflammation and atherosclerosis. *Circulation*.

[3] Libby P. (2002). Inflammation in atherosclerosis. *Nature*.

[4] Ammirati E., Moroni F., Norata G. D., Magnoni M., Camici P. G. (2015). Markers of Inflammation Associated with Plaque Progression and Instability in Patients with Carotid Atherosclerosis. *Mediators of Inflammation*.

[5] den Hartog A. G., Achterberg S., Moll F. L. (2013). Asymptomatic carotid artery stenosis and the risk of ischemic stroke according to subtype in patients with clinical manifest arterial disease. *Stroke*.

[6] Norris J. W., Zhu C. Z., Bornstein N. M., Chambers B. R. (1991). Vascular risks of asymptomatic carotid stenosis. *Stroke*.

[7] Inzitari D., Eliasziw M., Gates P. (2000). The causes and risk of stroke in patients with asymptomatic internal-carotid-artery stenosis. North American Symptomatic Carotid Endarterectomy Trial Collaborators. *The New England Journal of Medicine*.

[8] Harris T. B., Ferrucci L., Tracy R. P. (1999). Associations of elevated interleukin-6 and C-reactive protein levels with mortality in the elderly. *American Journal of Medicine*.

[9] Ridker P. M., Hennekens C. H., Buring J. E., Rifai N. (2000). C-reactive protein and other markers of inflammation in the prediction of cardiovascular disease in women. *The New England Journal of Medicine*.

[10] Ridker P. M., Rifai N., Stampfer M. J., Hennekens C. H. (2000). Plasma concentration of interleukin-6 and the risk of future myocardial infarction among apparently healthy men. *Circulation*.

[11] Blankenberg S., Luc G., Ducimetière P. (2003). Interleukin-18 and the risk of coronary heart disease in european men: the prospective epidemiological study of myocardial infarction (PRIME). *Circulation*.

[12] Koenig W., Khuseyinova N. (2007). Biomarkers of atherosclerotic plaque instability and rupture. *Arteriosclerosis, Thrombosis, and Vascular Biology*.

[13] Choudhary S., Higgins C. L., Chen I. Y. (2006). Quantitation and localization of matrix metalloproteinases and their inhibitors in human carotid endarterectomy tissues. *Arteriosclerosis, Thrombosis, and Vascular Biology*.

[14] Golledge J., McCann M., Mangan S., Lam A., Karan M. (2004). Osteoprotegerin and osteopontin are expressed at high concentrations within symptomatic carotid atherosclerosis. *Stroke*.

[15] Mallat Z., Corbaz A., Scoazec A. (2001). Expression of interleukin-18 in human atherosclerotic plaques and relation to plaque instability. *Circulation*.

[16] Hobeika M. J., Thompson R. W., Muhs B. E., Brooks P. C., Gagne P. J. (2007). Matrix metalloproteinases in peripheral vascular disease. *Journal of Vascular Surgery*.

[17] Cavusoglu E., Ruwende C., Chopra V. (2006). Tissue inhibitor of metalloproteinase-1 (TIMP-1) is an independent predictor of all-cause mortality, cardiac mortality, and myocardial infarction. *American Heart Journal*.

[18] Hwang S.-J., Ballantyne C. M., Sharrett A. R. (1997). Circulating adhesion molecules VCAM-1, ICAM-1, and E-selectin in carotid atherosclerosis and incident coronary heart disease cases: the Atherosclerosis Risk in Communities (ARIC) study. *Circulation*.

[19] Kato R., Momiyama Y., Ohmori R. (2006). High plasma levels of osteopontin in patients with restenosis after percutaneous coronary intervention. *Arteriosclerosis, Thrombosis, and Vascular Biology*.

[20] Hellings W. E., Moll F. L., De Vries J.-P. P. M. (2008). Atherosclerotic plaque composition and occurrence of restenosis after carotid endarterectomy. *Journal of the American Medical Association*.

[21] Van Lammeren G. W., Den Ruijter H. M., Vrijenhoek J. E. P. (2014). Time-dependent changes in atherosclerotic plaque composition in patients undergoing carotid surgery. *Circulation*.

[22] de Kleijn D. P. V., Moll F. L., Hellings W. E. (2010). Local atherosclerotic plaques are a source of prognostic biomarkers for adverse cardiovascular events. *Arteriosclerosis, Thrombosis, and Vascular Biology*.

[23] Toole J. F. (1996). ACAS recommendations for carotid endarterectomy. *The Lancet*.

[24] Coyne T. J., Wallace M. C. (1994). Surgical Referral for Carotid Artery Stenosis — The Influence of NASCET. *Canadian Journal of Neurological Sciences / Journal Canadien des Sciences Neurologiques*.

[25] Sigala F., Georgopoulos S., Papalambros E. (2006). Heregulin, Cysteine Rich-61 and Matrix Metalloproteinase 9 Expression in Human Carotid Atherosclerotic Plaques: Relationship with Clinical Data. *European Journal of Vascular and Endovascular Surgery*.

[26] Bradford M. M. (1976). A rapid and sensitive method for the quantitation of microgram quantities of protein utilizing the principle of protein dye binding. *Analytical Biochemistry*.

[27] Laemmli U. K. (1970). Cleavage of structural proteins during the assembly of the head of bacteriophage T4. *Nature*.

[28] Towbin H., Staehelin T., Gordon J. (1979). Electrophoretic transfer of proteins from polyacrylamide gels to nitrocellulose sheets: procedure and some applications. *Proceedings of the National Acadamy of Sciences of the United States of America*.

[29] Filis K. A., Arko F. R., Johnson B. L. (2002). Duplex ultrasound criteria for defining the severity of carotid stenosis. *Annals of Vascular Surgery*.

[30] Galis Z. S., Sukhova G. K., Lark M. W., Libby P. (1994). Increased expression of matrix metalloproteinases and matrix degrading activity in vulnerable regions of human atherosclerotic plaques. *The Journal of Clinical Investigation*.

[31] Panayiotou A. G., Griffin M. B., Tyllis T. (2013). Association of genotypes at the matrix metalloproteinase (MMP) loci with carotid IMT and presence of carotid and femoral atherosclerotic plaques. *Vascular Medicine (United Kingdom)*.

[32] Lin R.-T., Chen C.-H., Tsai P.-C., Ho B.-L., Hank Juo S.-H., Lin H.-F. (2012). Sex-specific effect of matrix Metalloproteinase-9 functional promoter polymorphism on carotid artery stiffness. *Atherosclerosis*.

[33] Ohmori R., Momiyama Y., Taniguchi H. (2003). Plasma osteopontin levels are associated with the presence and extent of coronary artery disease. *Atherosclerosis*.

[34] Wolak T. (2014). Osteopontin - A multi-modal marker and mediator in atherosclerotic vascular disease. *Atherosclerosis*.

[35] Steitz S. A., Speer M. Y., McKee M. D. (2002). Osteopontin inhibits mineral deposition and promotes regression of ectopic calcification. *The American Journal of Pathology*.

[36] Ward L. D., Hammacher A., Howlett G. J. (1996). Influence of Inerleukin-6. Dimerization on formation of the high affinity hexameric IL-6 receptor complex. *The Journal of Biological Chemistry*.

[37] Varghese J. N., Moritz R. L., Lou M.-Z. (2002). Structure of the extracellular domains of the human interleukin-6 receptor *α*-chain. *Proceedings of the National Acadamy of Sciences of the United States of America*.

[38] Yamagami H., Kitagawa K., Nagai Y. (2004). Higher levels of interleukin-6 are associated with lower echogenicity of carotid artery plaques. *Stroke*.

[39] Sprague A. H., Khalil R. A. (2009). Inflammatory cytokines in vascular dysfunction and vascular disease. *Biochemical Pharmacology*.

[40] Elkind M. S., Cheng J., Boden-Albala B. (2002). Tumor necrosis factor receptor levels are associated with carotid atherosclerosis. *Stroke*.

[41] Ota H., Reeves M. J., Zhu D. C. (2010). Sex differences in patients with asymptomatic carotid atherosclerotic plaque: In vivo 3.0-T magnetic resonance study. *Stroke*.

[42] Redgrave J. N. E., Lovett J. K., Rothwell P. M. (2010). Histological features of symptomatic carotid plaques in relation to age and smoking: The Oxford plaque study. *Stroke*.

